# Cost-Effectiveness of Annual Screening for Tuberculosis among Italian Healthcare Workers: A Retrospective Study

**DOI:** 10.3390/ijerph17051697

**Published:** 2020-03-05

**Authors:** Luca Coppeta, Giuseppina Somma, Savino Baldi, Elisabetta Tursi, Iacopo D’Alessandro, Andrea Torrente, Stefano Perrone, Antonio Pietroiusti

**Affiliations:** Department of Biomedicine and Prevention, University of Rome “Tor Vergata”, Viale Montpellier 1, 00185 Roma, Italy; giuseppina.somma@ptvonline.it (G.S.); savino.baldi@hotmail.it (S.B.); elisabetta.tursi@yahoo.it (E.T.); iacopodalessandro@libero.it (I.D.); andrea.torrente@yahoo.it (A.T.); stefano.perrone@ptvonline.it (S.P.); pietroiu@uniroma2.it (A.P.)

**Keywords:** latent tuberculosis, contact screening, Quantiferon, occupational health

## Abstract

Background. In the past few years, healthcare workers (HCWs) have been considered at higher risk for tuberculosis (TB) infection than the general population. On the other hand, recent studies have reported a low conversion rate among these workers. Recently, the Center for Disease Control (CDC) updated its recommendations, suggesting that an annual screening should not be performed in the absence of a documented exposure but only in workers with high-risk duties or with job tasks in settings at high risk of tuberculosis contagion (e.g., departments of infectious or pulmonary diseases). In fact, some studies showed that annual tuberculosis screening for all the HCWs was not cost-effective in countries with a low incidence of TB. In this study, we evaluated the conversion rate and the cost-effectiveness of two different tuberculosis screening strategies in a large population of Italian HCWs. Methods. In our retrospective study, we reviewed data coming from a tuberculosis screening conducted on 1451 HCWs in a teaching hospital of Rome. All workers were evaluated annually by means of the Quantiferon test (QFT) for a five-year period. Then, the conversion rate was calculated. Results. We found a cumulative conversion rate of 0.6%. Considering the cost of the QFT test (48.26 euros per person), the screening of the HCWs resulted in a high financial burden (38,902.90 euros per seroconversion). Only one seroconversion would have been missed by applying the CDC updated recommendations, with a relevant drop of the costs: 6756.40 euros per seroconversion, with a global save of 296,075.10 euros. Conclusion: The risk of TB conversion among our study population was extremely low and it was related to the risk classification of the setting. Giving these results, the annual tuberculosis screening appeared to not be cost effective. We conclude that a targeted screening would be a better alternative in HCWs with a higher risk of TB exposure.

## 1. Introduction

Tuberculosis (TB) transmission in a healthcare environment is a major public health concern. Healthcare workers (HCWs) are considered at higher risk for TB contagion compared to the general population, due to their possible occupational exposure to *Mycobacterium tuberculosis* [1,2]. For HCWs in intermediate to high-risk setting, the annual probability of exposure to contagious TB patients is estimated to range between 1.3% and 13.5%, depending on both the working area and the job task [3,4,5]. After exposure to a contagious TB patient, HCWs have a 22% probability of becoming infected, but this percentage is highly variable depending on many factors (i.e., characteristics of the patient, employee immune status, etc.) [5]. In a recent meta-analysis, HCWs have been estimated to be both at high risk for active (A) and latent tuberculosis infection (LTBI) based on the results of epidemiological studies conducted in high and intermediate incidence countries [2]. A follow-up study carried out among a large number of North American hospital workers during the period 1995–2007 reported that TB incidence rates among HCWs were similar to those of the general population [6], foreign birth being the major risk factor of LTBI [7,8,9,10,11,12,13]. Moreover, a recent retrospective cohort study found an extremely low rate of Tuberculin Skin Test (TST) conversion among a large population of HCWs working in the low TB-incidence United States. In this population, the observed conversion rate was 0.3% per year, and a limited proportion of cases was attributable to occupational exposure [14], raising questions about the cost-effectiveness of routine screening for TB among HCWs. A recent analysis conducted in Canada found that the annual TST screening strategy was not cost effective when compared with more targeted TST screening [15]. The annual TST screening strategy yielded an extremely high incremental cost per additional case prevented versus targeted TST screening. Based on the results of those studies, the Center for Disease Control (CDC) recently updated the TB screening recommendations for HCWs [16]. These recommendations stated that in the absence of known exposure or evidence of ongoing TB transmission, U.S. HCWs should not undergo routine serial testing at any interval after baseline evaluation, except for certain occupational groups who might be at increased risk for TB transmission (e.g., pulmonologists or respiratory therapists) or for those HCWs working in settings where the transmission has occurred in the past (e.g., emergency departments) [16]. The main reasons for those recommendations were: the low risk of TB transmission among HCWs, the poor sensitivity and specificity of available screening tests (TST and Interferon-Gamma Release Assays/IGRAs) in low-risk groups and the unfavorable cost-effectiveness of serial screening [12,17]. In Italy, TB transmission is actually low, being 6.5/10000 per year, of which most of the cases regarding foreign persons come from high incidence countries [18]. Actually, based on the Italian Ministerial recommendations, TB screening for HCWs is performed at baseline, after known exposure, and periodically (most often annually) in the intermediate to high-risk settings [19]. Our study aimed to evaluate the TB conversion rate among a group of HCWs in Italy employed at a university hospital in Rome and the cost-effectiveness of annual screening in those subjects, as compared to the results and costs that would had been obtained by applying the updated CDC recommendations.

## 2. Materials and Methods

In our retrospective study, we reviewed the clinical records of all HCWs employed at the Tor Vergata University hospital, whether or not working in high TB risk areas. Data were collected during the health surveillance visit at the Occupational Medicine Unit throughout the period from 1 January 2013 to 31 December 2018. TB infection was evaluated through the Quantiferon test (QFT). The results of the test were classified in accordance with the interpretative guidance provided by the manufacturer, i.e., “positive” or “negative” are respectively classified whether above or below the cutoff level of 0.35 IU/ml for antigen-specific Interferon-Gamma when compared to a negative control.

Conversion was defined as a positive test in a previously negative subject. Latent tuberculosis infection condition is defined by the positivity of the QFT test and the negativity of the clinical–radiological assessment, which was conducted for excluding an active infection. For each subject, the following data were recorded: age at the beginning of the study, gender, job task, and area of employment. The risk classification for each subject enrolled was performed according to the 2013 Italian guidelines. These guidelines encompass five growing levels from A (low risk) to E (ongoing transmission) based on the risk evaluation at area, department, and single operator level.

Inclusion criteria: HCWs negative at the baseline test (at the beginning of observational period) and having a job task including the direct patient care (medical doctors, nurses, and laboratory and radiology technicians). Exclusion criteria: HCWs with a positive test at baseline, having previous diagnosis or treatment for active and/or latent TB infection, performing only administrative tasks or other tasks not involving the direct patient care and finally, HCWs with incomplete records or with a long (>12 months) period of absence from work during the observational period.

We evaluated the conversion rate among our study population in respect to the more significant variables using univariate and multivariate regression analysis.

Moreover, we compared the cost-effectiveness of the current annual serial screening practices for LTBI among Italian HCWs working in a setting where they may meet TB patients, with that of a “targeted” test approach recommended by CDC 2019 updates. According to this strategy, only operators involved in high-risk activities (pneumologists or workers in an emergency area) and/or recent contact with a case of active disease should be tested. For cost–benefit analysis, we assumed a 100% compliance of HCWs to the screening test for both strategies. In the annual screening strategy, all subjects negative at baseline were retested with QFT during the subsequent year, regardless of their risk profile. In targeted screening strategy, we considered that HCWs negative at baseline were retested only after recognized exposure to a case of contagious TB and/or annually if they were employed in high-risk areas.

**Figure 1:**
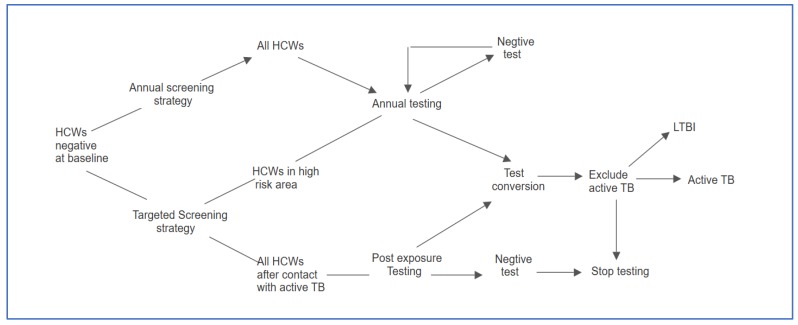
Representation of the two screening strategies. HCWs: healthcare workers, TB: tuberculosis, LTBI: latent TB infection.

Operators who converted their QFT test from negative to positive underwent investigation for active TB and were offered isoniazid for LTBI, once active disease was excluded. Moreover, those workers were excluded from subsequent QFT tests (Figure 1).

The annual risk of progression to active TB for infected workers was estimated to be 10% lifetime [1]. Since CDC guidelines suggest using Interferon-Gamma Release Assays for the diagnosis of latent tuberculosis infection among HCWs [20], we used the direct cost of the QFT test (48.26 euros) in the cost-effectiveness evaluation [21].

The data were processed with IBM SPSS statistical package release 21.

The study was approved by the ethical committee of the Policlinic of Rome Tor Vergata. Ethical committee authorization number 194/2018.

## 3. Results

We examined the clinical records of 1451 subjects (478 males and 973 females). The mean age was 40.9 years. The main characteristics of the study populations are shown in Table 1.

Conversion at the QFT test was found in 9/1471 subjects (0.6%) during the whole study period corresponding to a rate of 1.2/1000 per year. The rate of conversion was higher among male (1.2% versus 0.3%; *p* < 0.005), subjects over 40 years old (0.8% versus 0.4%; *p* = n.s.), nurses (0.9% versus 0.4%), workers in high-risk settings (1.3% versus 0.5%; *p* = n.s.) and occupation group recommended for annual screening according to CDC 2019 guidelines (3.6% versus 0.1%; *p* < 0.001).

The odds ratio (OR) for IGRA conversion over the five-year period, in relation to gender, age class, job task, risk class, and suitability to be included in the periodical follow up according to CDC 2019 recommendations are reported in Table 2. We found that the male gender and CDC classification were the only relevant predicting variables in the multivariate analysis. The annual conversion rate was 7.0/1000 in the group for whom the screening is recommended and 0.2/1000 in the group not suitable for routine screening according to CDC recommendations. Only one case of conversion occurred in the last group during the observational period.

No case of active TB was found in the study population during the follow-up.

Regarding the cost analysis, we compared the cost-effectiveness of the two hypotheticals strategies of screening (annual testing versus CDC “targeted” screening). The annual screening of the HCWs by means of the QFT test resulted in high costs (38,902.90 euros per seroconversion). Only one case of seroconversion would have been missed by applying the CDC updated recommendations, with a relevant drop of the costs: 6756.40 euros per seroconversion. Its application would have saved about 296.075 euros among our population in the study period. Considering the effective conversion rate and the risk of progression reported in the literature (10% lifetime) [1], we should expect to have no case of active tuberculosis in the next 100 years; moreover, the annual screening strategy using the IGRA test should have yielded an incremental cost estimate respectively of 2960.750 euros per additional case prevented versus targeted screening.

## 4. Discussion

HCWs have been historically considered to be at higher risk for tuberculosis infection, but it could be no longer the case [14,15,16]. Our study suggests that, in an Italian hospital setting, the conversion rate is low, and the routine annual screening for HCWs who work in typical healthcare facilities provides limited benefit at high cost as compared to a more targeted strategy. The recent updated recommendations from the National Tuberculosis Controllers Association and Center for Disease Control for tuberculosis screening, testing, and treatment of HCWs in the United States suggest testing all HCWs at baseline with IGRA (or TST in alternative) but not to perform routine serial TB testing at any interval after baseline in the absence of a known exposure or ongoing transmission [16]. Moreover, according to those guidelines, serial TB testing should involve groups of workers who might be considered at higher occupational risk for TB (such as pulmonologists) or workers in certain areas where the transmission has occurred in the past, such as the emergency departments. The rationale for those recommendations (low conversion rate among US hospital personnel, limited sensitivity and specificity of both IGRA and TST in low-incidence populations) seems to be valid in our population. Testing low-risk HCWs in our study led to a limited benefit if any with large incremental costs. The application of CDC recommendations would have allowed us to identify all but one case of conversion in the study period.

Considering the conversion rate in workers not included according to the CDC recommendations and the rate of conversion among immunocompetent HCWs, the overall expected risk to develop an acute TB case in our population is of one case during the next 50 years period, which is a risk that can be considered negligible. Nevertheless, the estimated cost of preventing those additional cases of TB in our setting is surprisingly high, and the annual screening seems not to be justified. Indeed, the resources used for the testing could be spent more effectively in the prevention of occupational contagion in intermediate/high risk groups. In a study conducted in Canada [15], the annual TST screening strategy was not cost-effective and yielded an additional cost estimate of $1717.539 Canadian dollars (over 1,511,000 euros) per additional case prevented versus targeted screening. Those evaluations were based on the conversion rate estimated in a low-risk scenario, as it is in our case.

Moreover, false positive subjects would have received unnecessary radiological screening, unnecessary six-months isoniazid therapy with related indirect costs (estimated to be over 1400 euros), and potential health risk due to the hepatotoxicity of the antibiotic regimen [22].In Italy, there is not a mandatory regulation regarding the frequency of routine test in the hospital population. However, in intermediate to high-risk settings, it is usually performed annually. Our study clearly shows that the CDC update recommendations are applicable to the Italian HCWs and are highly cost-effective.

A reasonable alternative strategy to the use of the IGRA test on a targeted identified population at higher risk of TB infection is the use of TST as a first-step screening, according to the Italian guidelines [19], in particular for those HCWs undergoing serial testing. According to this strategy, the IGRA test has a central role as a TB second step-screening, in order to reduce the number of people that should undergo more invasive tests (X-ray diagnosis and/or chemotherapy). TST followed by QFT was particularly cost-effective when the rate of LTBI in the screened population was low [23,24].

## 5. Conclusions

Our study shows that the risk of TB conversion among an Italian hospital population is extremely low and it is related to the risk classification of the setting. Giving these results, the annual tuberculosis screening for all the HCWs appeared to not be cost-effective, according to the 2019 CDC guidelines. Giving this data, we may conclude that a targeted screening would be a better alternative if conducted in HCWs with a higher risk of TB exposure.

## Figures and Tables

**Table 1:** Main characteristics of study population. IGRA: Interferon-Gamma Release Assays.

Variables	*n*	%
**Subjects**	1451	100
**Mean age**	40.95	
**Age class**		
≤40 years	733	50.5
>40 years	718	49.5
**Gender**		
Males	478	33
Females	973	67
**Born in high incidence countries**		
No	1417	97.7
Yes	34	2.3
**Seniority**		
≤10 years	424	29
>10 years	1027	71
**Job task**		
Nurse	649	44.7
Medical doctor	555	38.2
Laboratory personnel	126	8.7
Technician	20	1.4
Dentistry	11	0.8
Other	90	6.2
**Risk level**		
Low-intermediate (Level A, B, and C)	1319	90.9
High risk (Level D, E)	132	9.1
**Periodical screening recommended (CDC 2019)**		
No	1227	84.6
Yes	224	15.4
**Conversion at IGRA test**		
No	1442	99.38
Yes	9	0.62

**Table 2:** Odds ratio (OR) for IGRA conversion in relation to gender, age class, job task, and risk classification.

Variables	O.R.	95% (C.I.)	*p*
Male gender	4.25	1.01–18.08	<0.05
Age >40 years	2.22	0.52–9.42	n.s.
Job task: nurse	0.61	0.13–2.88	n.s.
Working in high risk setting (Level D, E)	0.75	0.15–3.80	n.s.
Recommended for periodical screening	65.8	7.42–584.41	<0.01
